# Psychotherapy assisted by Ketamin: a narrative review

**DOI:** 10.1192/j.eurpsy.2026.11734

**Published:** 2026-07-31

**Authors:** V. Fournier, A. Kuntz, R. Bouzegaou, I. Gothuey

**Affiliations:** 1 Medical section, Fribourg University; 2 Addictions, Fribourg Mental Health Network and Fribourg University; 3 Addictions, Fribourg Mental Health Network; 4 Adult Psychiatry, Fribourg Mental Health Network and Fribourg University, Fribourg, Switzerland

## Abstract

**Introduction:**

As the number of treatment resistant patient in the psychiatric field has been growing over the last decades, there is a clear need for new therapeutic options. Over several years, the field of psychedelic assisted psychotherapy has been rapidly developing. In this scope ketamine assisted psychotherapy (KAP) has come into psychiatric use to treat depression resistant depression, generalized anxiety disorder, post-traumatic stress disorder as well as other psychiatric troubles.

**Objectives:**

The aim of this narrative review is to provide a better understanding of the KAP, its indications and the possibles changes during the course of the patient’s psychotherapy. In addition, this review studies the efficiency of the KAP and the possible associated risks.

**Methods:**

Extended research has been made on the PubMed and Embase database, reviewing the articles written between 2015 and 2025 using the keys and mesh words psychotherapy and ketamine. Inclusions criteria were the following: use of psychotherapy, KAP session should be done is a relaxing setting and use a bolus dose inducing a psychedelic experience. Twenty for three articles have been finally selected using these inclusion criteria and additional exclusions criteria.

**Results:**

This study shows the clear efficiency of the KAP in the treatment for resistant depression, generalized anxiety disorder, post-traumatic stress disorder as well as other psychiatric troubles as addictions at a short term. These results could be partly related to the positive changes in the patient functioning in his psychotherapy after living a psychedelic experience.

Nevertheless, a large part of patient, varying from 30 to 50%, are not responding to the therapy.

This study has also shown a low risk associated to the KAP when properly conducted, which underlines the need for clearer guidelines and medical good practices.

**Conclusions:**

KAP shows a good therapeutic potential and should yet carefully put into practice through clinical trials. Further studies are yet needed to better understand why some patients aren’t responding to the therapy and what could be done to improve the number of responding patients.

The long-term efficiency of KAP isn’t yet clear, which opens the door to new studies.

**Disclosure of Interest:**

None Declared

